# Dietary *Galla Chinensis* tannic acid supplementation in the diets improves growth performance, immune function and liver health status of broiler chicken

**DOI:** 10.3389/fvets.2022.1024430

**Published:** 2022-10-14

**Authors:** Jiaxing Niu, Qinjin Wang, Changwei Jing, Yang Liu, Hua Liu, Ning Jiao, Libo Huang, Shuzhen Jiang, Qinglin Guan, Yang Li, Weiren Yang

**Affiliations:** ^1^Shandong Provincial Key Laboratory of Animal Biotechnology and Disease Control and Prevention, Department of Animal Science and Veterinary Medicine, Shandong Agricultural University, Taian, China; ^2^Shandong Wonong Agro-tech Group Co., Ltd., Weifang, China; ^3^College of Animal Science and Technology, Hunan Agriculture University, Changsha, China; ^4^Shandong Landoff Biotechnology Co., Ltd., Taian, China

**Keywords:** broiler chicken, *Galla Chinensis*, immune function, liver health, tannic acid, TLR4/MyD88/NF-κB

## Abstract

This experiment was conducted to investigate the effects of *Galla Chinensis* tannic acid (TA) on growth performance, immune function, and liver health status in broilers. A total of 288 1-day-old Arbor Acres broiler chickens were randomly divided into two groups in a 42-days study. The two groups were a basal diet (CON group) and a basal diet supplemented with 300 mg/kg *Galla Chinensis* tannic acid (TA group). The results showed that the TA group had significantly decreased feed-to-gain ratio (F/G) throughout the experiment (*P* < 0.05). The levels of total protein, albumin, low density lipoprotein, high density lipoprotein, urea, total cholesterol, and glucose in the TA group were significantly higher than in the CON group (*P* < 0.05). In addition, the serum immunoglobulin G, immunoglobulin M, and complements (C3, C4) levels in the TA group were significantly higher than those in the CON group (*P* < 0.05). Compared with the CON group, the hepatic interleukin-6, interleukin-18, NLRs family pyrin domain containing 3 (NLRP3), caspase-1, and caspase-3 in the TA group were significantly decreased (*P* < 0.05). Besides, TA group had significantly lower mRNA expression levels of toll-like receptor 4 (*TLR4*), myeloid differentiation primary response 88 (*MyD88*), nuclear factor-kappa B (*NF-*κ*B*), and *NLRP3* in liver (*P* < 0.05). The TA group had significantly higher the mRNA expression levels of *Bcl-2* than CON group in liver (*P* < 0.05). Moreover, TA group tended to decrease *Bax/Bcl-2* ratio in liver (*P* < 0.10). To sum up, dietary supplemented with microencapsulated TA from *Galla Chinensis* had beneficial effects on growth performance, immune function, and liver health status in broilers. The protective role of TA from *Galla Chinensis* in liver health of broilers might be related to the inhibition of hepatic apoptosis and pyroptosis *via* inactivation of TLR4/MyD88/NF-κB signaling pathway.

## Introduction

The liver is not only one of the important metabolic organs, but also performs activities of immune function of the body (1, 2). Liver plays an important role in regulating the production and metabolism of fat, carbohydrate, and protein, and cytokines, complement components, chemokines, and is responsible for the synthesis of cytokines, complement components, chemokines, and acute phase proteins (3, 4). In addition, liver has specific functions in clearing pathogens and exogenous antigens in systemic circulation, detoxify, and phagocytize bacteria (5, 6). Recently, intensive poultry farming significantly boosts livestock productivity and profits, but it also raises the chance of liver damage due to pathogenic bacterium, environmental factors, and poor feed hygiene (7, 8), which adversely affecting the growth performance, liver health, and immunity of broilers. Therefore, liver protection is great significance to ensure the healthy and sustainable development of poultry production.

A huge number of studies illustrated that Chinese herbs extract had anti-inflammatory, antioxidant, and antimicrobial activities (9–11). Supplementation with Chinese herbs extract in broiler diets has been proven to improve growth performance and benefit liver health (11, 12). *Galla Chinensis* is one of the important Chinese herbs, and its main bioactive component is a hydrolysable tannic acid (TA) called gallotannins that is a glycoside or ester formed by the combination of gallic acid and diacid with glucose (13, 14). It was reported that TA has numerous biological functions such as anti-inflammatory, antioxidant, anti-bacterial, antiviral, and apoptosis regulation (15–18). However, tannins were once thought to be an anti-nutritional feed additive for monogastric animals due to their binding properties with proteins, carbohydrates, minerals, and digestive enzymes, which reduced bioavailability of nutrients in feed (19). Recent studies found that TA supplementation could improve growth performance, promote liver health, and enhance immune function of animals (20, 21), suggesting that TA could be a good feed additive for animal production. However, TA was generally derived from chestnut in majority, and few studies investigated the *Galla Chinensis* TA as the feed additive in poultry research. Wang, et al. (22) showed that TA from *Galla Chinensis* addition had beneficial effects on intestinal morphology, intestinal nutrient transporter, and intestinal microbiota in weaning piglets. Besides, our recent study also showed that supplementation of 300 mg/kg TA extracted from *Galla Chinensis* had beneficial effects on intestinal development and suppressed intestinal mucosal inflammatory responses in broilers (23). When the gut is damaged, it increases the translocation of harmful metabolites to the liver, leading to impaired liver function (24). However, little information is available on the evaluation of the effects of dietary *Galla Chinensis* TA supplementation on the liver health in broilers.

Therefore, the purpose of this experiment was to investigate the effects of *Galla Chinensis* TA on growth performance, immune function, and liver health status in chickens, which provided new insights into the use of *Galla Chinensis* TA as a new feed additive in poultry production.

## Materials and methods

### Animals, diets, and management

A total of 288 one-day-old AA broilers with an initial body weight (BW) of 48.05 ± 0.39 g were randomly divided into two treatment groups, each of which included six replicates of 24 broilers. The experiment lasted 42 days. The two experimental groups were as follows: (1) CON group, broilers fed a basal diet; (2) TA group, broilers fed a basal diet supplemented with 300 mg/kg TA (23). The TA production, extracted from *Galla Chinensis*, was microencapsulated with 40% effective concentrations and provided by the Wufeng Chicheng Biotechnology Co., Ltd (Yichang, China). The basal diets (Table 1) were formulated according to two-phase feeding programs (0–21 d and 21–42 d) recommended by Ministry of Agriculture of China (2004) (25). All broilers had free access to water and feed throughout the trial. Each replicate per group contained 24 broilers and housed in a three-level mental chicken coop placed in an environment-controlled room with continuous light, providing 5 lx of light intensity (26). During the experiment, daily feed intake and BW (on days 21 and 42) per replicate were recorded to calculate average daily feed intake (ADFI), average daily gain (ADG), and feed-to-gain ratio (F/G). The room temperature was maintained at 35°C for the first week and then decreased by 1°C every 2 days until 21°C.

**Table 1:** Ingredients composition and nutrient levels of basal diets (as-fed basis).

**Items**	**Phases**	
	**0–21 d**	**21–42 d**
**Ingredients, %**		
Corn	55.91	55.91
Soybean meal, 44% CP	13.78	10.18
Wheat bran	11.98	12.98
Corn starch residue	7.99	9.98
Corn gluten meal	3.99	3.99
Extruded soybean	1.50	2.10
Limestone	1.70	1.70
Calcium monophosphate	1.10	1.10
L-Lysine HCl, 76.8%	1.00	1.00
DL-Methionine, 98%	0.20	0.20
L-Threonine, 98%	0.10	0.10
Sodium chloride	0.40	0.40
Choline	0.10	0.10
Phytase	0.10	0.10
Complex enzyme	0.02	0.02
Trace mineral premix^1^	0.10	0.10
Vitamin premix^2^	0.02	0.02
Antioxidant	0.02	0.02
Total	100	100
**Nutrient content** ^**3**^ **%**		
Metabolizable energy, MJ/kg	12.33	12.50
Crude protein	19.47	17.93
Crude fat	3.45	3.74
Calcium	0.94	0.87
Available phosphorus	0.35	0.33
Lysine	1.15	1.00
Methionine	0.50	0.40

^1^Provided per Kilogram of Complete Basal Diet: 10 mg of Cu as CuSO4, 100 mg of Fe as FeSO4, 1.1 mg of I as Ca(IO3)2, 65 mg of Zn as ZnSO4, 100 mg of Mn as MnSO4 and 0.3 mg of Se as Na2SeO3.

^2^Provided per Kilogram of Complete Basal Diet: Vitamin A 10,000 IU, Vitamin D3 3,000 IU, Vitamin E 30 IU, Menadione 1.3 mg, Thiamine 2.2 mg, Riboflavin 8 mg, Pyridoxine 4 mg, Vitamin B12 0.025 mg, D-Biotin 0.2 mg, Niacin 40 mg, Folic Acid 1 mg and D-Calcium Pantothenate 10 mg.

^3^The contents of crude protein, crude fat, calcium and available phosphorus in basal diets were analyzed according to the AOAC method of analysis. Others are calculated according to the tables of feed composition and nutritive in China (2019).

### Sample collection

On the morning of day 42, six chickens (one chicken each replicate) were randomly selected from each group. Fasting blood was collected from the wing vein using an evacuated blood collection tube. Then serum was harvested by being centrifuged at 3,500 r/min for 15 min, and stored at −20°C until further analysis. After evisceration, livers were weighed to calculate the ratio of liver weight to BW individually. Besides, 5 g of liver samples were quickly collected from the middle of hepatic lobule. One part of the liver samples was stored at −80°C refrigerator until further analysis after being quick-frozen with liquid nitrogen, and another part was fixed with 4% paraformaldehyde solution for 24 h at room temperature.

### Analysis of liver morphological sections

After fixation with 4% paraformaldehyde solution for 24 h, liver samples were dehydrated through graded levels of ethyl alcohol (70, 80, 85, 90, 95, 100 I, and 100% II) and embedded in liquid paraffin according to the standard histological procedure (27). Then, paraffin wax embedded liver tissues were cut into 5 μm sections and stained with hematoxylin and eosin (H&E). Finally, liver sections were observed under an Olympus B X 51 microscope (Tokyo, Japan).

### Analysis of concentrations of serum biochemical indicators

After the preserved serum samples were dissolved at 4°C, serum levels of albumin (ALB), total protein (TP), high density lipoprotein (HDL), urea, triglycerides (TG), total cholesterol (TCHO), low density lipoprotein (LDL), glucose (GLU), and alanine aminotransferase (ALT) were determined by using the kits purchased from Nanjing Jiancheng Bioengineering Institute (Nanjing, China) on a COBUS MIRA Plus automatic biochemical analyzer (Roche Diagnostic System Inc, United States).

### Analysis of immunoglobulins and complements concentrations in serum

Serum concentrations of immunoglobulin G (IgG), immunoglobulin A (IgA), immunoglobulin M (IgM), and complements (C3 and C4) were detected with commercial ELISA kits (Jiangsu Meimian Industrial Co., Ltd, Jiangsu, China) on the basis of the procedure previously described (28).

### Analysis of liver function parameters and inflammatory factors in livers

The concentrations of NLRs family pyrin domain containing 3 (NLRP3), tumor necrosis factor-α (TNF-α), interleukin-1β (IL-1β), interleukin-6 (IL-6), interleukin-18 (IL-18), caspase-1, and caspase-3 in liver were measured using a commercial assay kit (R&D Systems Inc., Minneapolis, MN, United States) derived from a previous study (29).

### RNA isolation, cDNA synthesis, and qPCR

Liver samples were ground in liquid nitrogen and total RNA from the liver samples was extracted using a TRIzon Reagent RNA kit according to the manufacturer's instructions (Accurate Biology, Hunan, China). Equal mass of RNA of each sample was reverse transcribed into cDNA using reverse transcription (RT) kit (Accurate Biology, Hunan, China). The qRT-PCR was performed using SYBR Green Premix Pro Taq HS qPCR Kit (AG11701, Accurate Biology, DaLian, China) and gene specific primers (myeloid differentiation primary response 88, NLRs family pyrin domain containing 3, nuclear factor-kappa B, toll-like Receptor 4, B-cell lymphoma-2, and BCL2-Associated X) according to manufacturer's instruction. Gene expression relative to reference gene β*-actin* was calculated by 2^−ΔΔCT^ method. Gene specific primers are shown in Table 2.

**Table 2:** Primer sequences used for fluorescent quantitation PCR.

**Genes ^2^**	**Genbank**	**Primer sequences, 5^′^-3^′1^**	**Size, bp**
*β-actin*	NM_205518.1	F:ATTGTCCACCGCAAATGCTTC R:AAATAAAGCCATGCCAATCTCGTC	113
*TLR4*	NM_001030693.2	F:CATCTCTGGAGTTCCTGCTGAA R:TGTATGGATGTGGCACCTTGA	145
*MyD88*	NM_001030962.5	F:CGGAGGATGGTGGTCGTCATT R:TCGTTCTTCATGGTCTTGCACTTG	140
*NF-κB*	NM_001396038.1	F:CAGCCCATCTATGACAACCG R:TCAGCCCAGAAACGAACCTC	152
*NLRP3*	NM_001348947.2	F:GAAGGTGCTGCTATGGACATTG R:CGTGCTCTGTGTATTTCTGCTTAT	118
*Bax*	XM_422067	F:TGAGCATGTAGCAACGGAAG R:AGCAAGCTGATTGACGGTCT	295
*Bcl-2*	NM_205339.3	F:AGGACAACGGAGGATGGGATG R:CACCAGAACCAGGCTCAGGAT	110

^1^F, Forward primer; R, Reverse primer.

^2^TLR4, Toll-Like Receptor 4; MyD88, Myeloid Differentiation Primary Response 88; NF-?B, Nuclear Factor Kappa-Light-Chain-Enhancer of Activated B Cells; NLRP3, nod Like Receptor Family Pyrin Domain Containing 3; Bax, BCL2-Associated X; Bcl-2, B-Cell Lymphoma-2.

### Statistical analysis

The replicate was considered the experimental unit for the analysis of growth performance, and individual broiler was considered the experimental unit for all other variables. The *t*-test procedure of SAS 9.4 software (Institute Inc., Cary, NC, United States) was used to evaluate the significance between the CON group and the TA group after assessing the normality of data. The results were expressed as means ± standard error (SE). Significant differences were indicated by ^*^P < 0.05, ^**^P < 0.01, and ^***^P < 0.001, while ^#^0.05 ≤ *P* < 0.1 was indicated as a significant tendency.

## Results

### Effect of TA on growth performance of broilers

As presented in Table 3, from d 21 to 42 and from d 0 to 42, broilers in TA group had significantly lower (*P* < 0.05) F/G than broilers in CON group, and the ADG in TA group tended to be higher (*P* < 0.10) than that in CON group. Besides, the BW in TA group tended to be higher (*P* < 0.10) than that in CON group on d 42, and TA group tended to be higher (*P* < 0.10) ADFI than CON group from d 0 to 21.

**Table 3:** Effects of dietary supplemented with microencapsulated TA from *Galla Chinensis* on growth performance of broiler chickens.

**Items**	**CON**	**TA**	***P*-value**
**BW, g**			
d 0	47.60 ± 0.83	48.49 ± 0.84	0.478
d 21	775.70 ± 7.62	790.28 ± 7.85	0.231
d 42	2171.33 ± 4.47	2336.28 ± 64.95	0.084
**ADFI, g**			
d 0–21	57.32 ± 0.99	59.80 ± 0.43	0.060
d 21–42	142.12 ± 1.92	140.25 ± 3.98	0.688
d 0–42	99.72 ± 1.12	100.03 ± 1.92	0.889
**ADG, g**			
d 0–21	34.67 ± 0.39	35.32 ± 0.38	0.280
d 21–42	66.46 ± 0.43	73.62 ± 2.89	0.088
d 0–42	50.57 ± 0.11	54.47 ± 1.56	0.087
**F/G**			
d 0–21	1.66 ± 0.02	1.69 ± 0.03	0.287
d 21–42	2.14 ± 0.04	1.91 ± 0.03	0.005
d 0–42	1.90 ± 0.02	1.80 ± 0.02	0.018

CON, broiler chickens fed a basal diet; TA, broiler chickens fed a basal diet supplemented with 300 mg/kg microencapsulated TA from Galla Chinensis. BW, body weight; ADFI, average daily feed intake; ADG, average daily gain; F/G, feed-to-gain ratio. Values are mean ± standard error (n = 6).

### Effects of TA on serum metabolites of broilers

**Figure 1:**
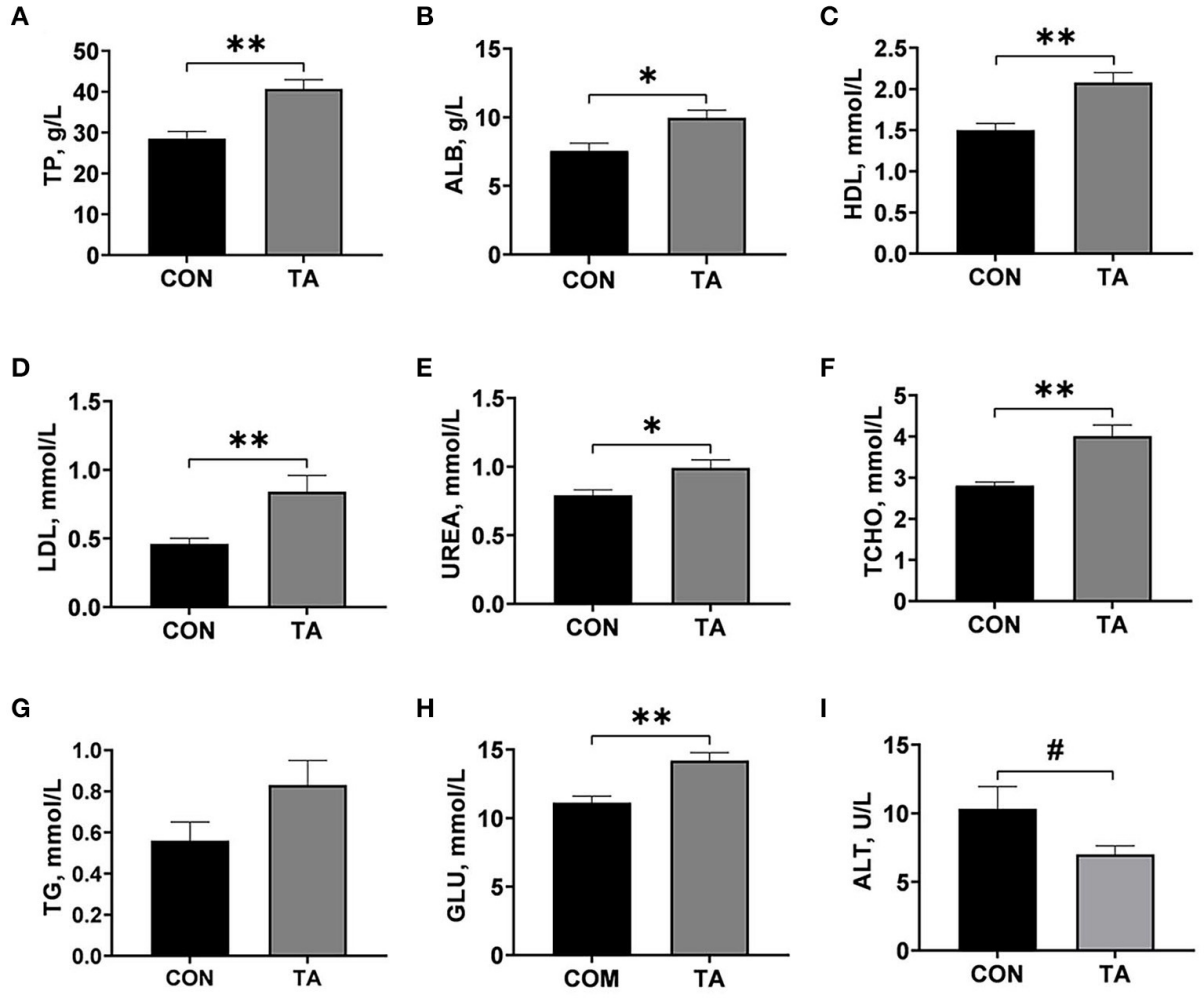
Effects of dietary supplemented with microencapsulated TA from *Galla Chinensis* on serum biochemical parameters of broiler chickens. **(A)** Total protein (TP), **(B)** Albumin (ALB), **(C)** High density lipoprotein (HDL), **(D)** Low density lipoprotein (LDL), **(E)** UREA, **(F)** Total cholesterol (TCHO), **(G)** Triglycerides (TG), **(H)** Glucose (GLU), **(I)** Alanine transaminase (ALT). CON, broiler chickens fed a basal diet; TA, broiler chickens fed a basal diet supplemented with 300 mg/kg microencapsulated TA from *Galla Chinensis*. Values are mean ± standard error (*n* = 6). *P < 0.05, **P < 0.01, ^#^0.05 < *P* < 0.1.

Serum metabolites concentrations of broilers on day 42 were presented in Figure 1. The serum TP, ALB, HDL, UREA, TCHO, LDL, and GLU concentrations in broilers fed TA diet were significantly higher (*P* < 0.05) than those in broilers fed CON diet. In contrast, the TA group tended to have lower (*P* < 0.10) ALT activity than the CON group. No significant difference (*P* > 0.05) was observed in TG concentration between the CON group and the TA group.

### Effects of TA on serum immunoglobulins concentrations and completes concentrations of broilers

**Figure 2:**
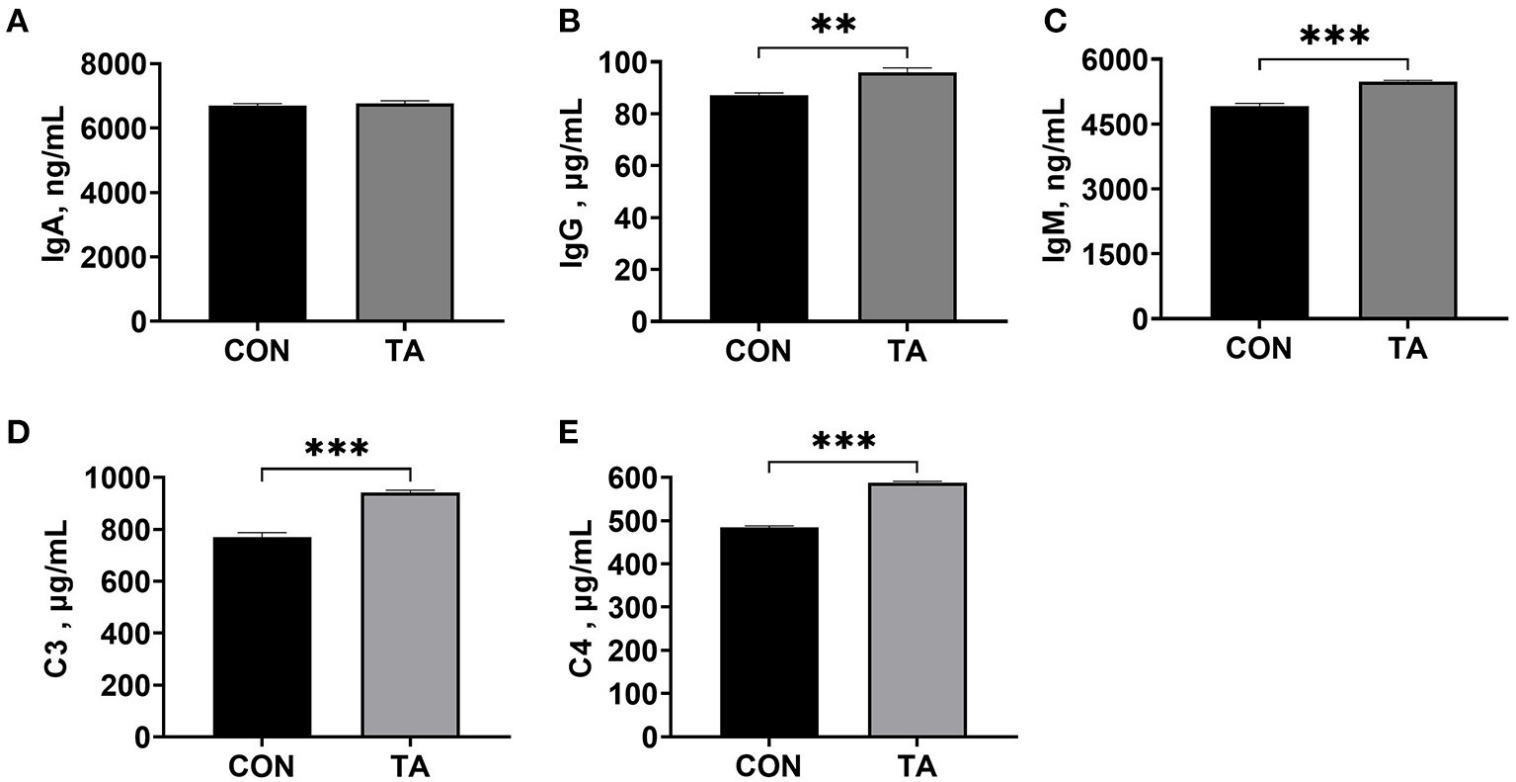
Effects of dietary supplemented with microencapsulated TA from *Galla Chinensis* on serum immunoglobulins and complements concentrations of broiler chickens. **(A)** Immunoglobulin A (IgA), **(B)** Immunoglobulin G (IgG), **(C)** Immunoglobulin G (IgG), **(D)** Complement C3, **(E)** Complement C4. CON, broiler chickens fed a basal diet; TA, broiler chickens fed a basal diet supplemented with 300 mg/kg microencapsulated TA from *Galla Chinensis*. Values are mean ± standard error (*n* = 6). **P < 0.01, ***P < 0.001.

As shown in Figure 2, the serum concentrations of IgG, IgM, C3, and C4 were significantly increased (*P* < 0.05) in TA group than the CON group. There was no significant difference (*P* > 0.05) in serum IgA levels between the CON and TA groups.

### Effects of TA on liver histolomorph and index of broilers

**Figure 3:**
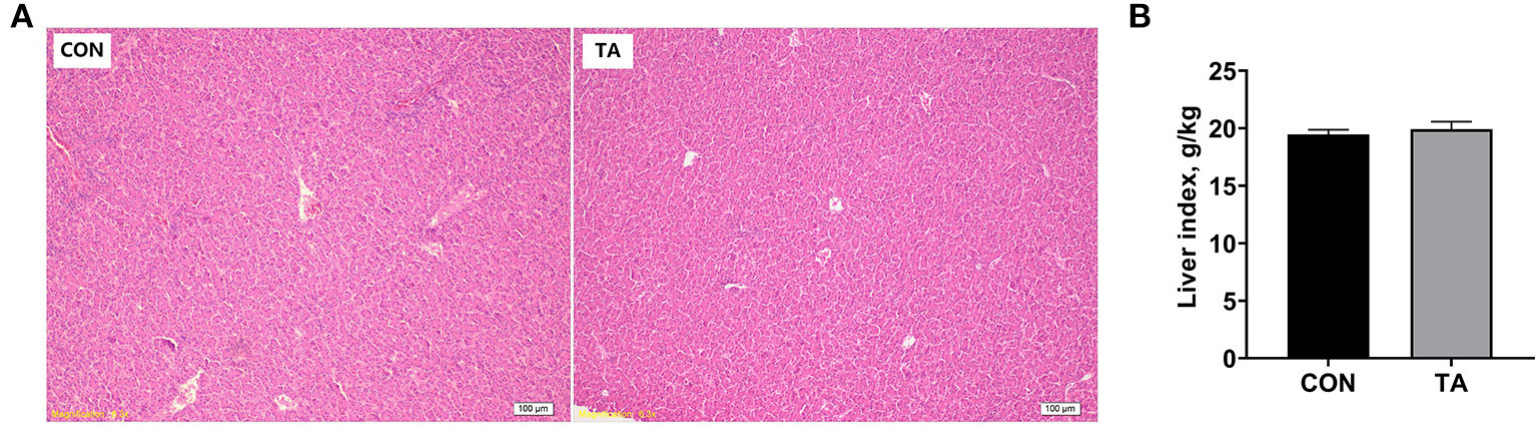
Effects of dietary supplemented with microencapsulated TA from *Galla Chinensis* on liver morphological sections and liver index of broiler chickens. **(A)** Liver morphological sections, **(B)** Liver index (g/kg) = liver weight (g)/body weight (kg). CON, broiler chickens fed a basal diet; TA, broiler chickens fed a basal diet supplemented with 300 mg/kg microencapsulated TA from *Galla Chinens*is. Values are mean ± standard error (*n* = 6).

The liver histolomorph and index are shown in Figure 3. Compared with the CON group, the liver cells were structurally complete and arranged in an orderly manner in TA group. Moreover, TA group showed lower degrees of liver inflammatory cell infiltration than CON group. There was no significant difference (*P* > 0.05) in liver index between the two groups.

### Effects of TA on inflammatory factors concentrations and caspases activities in liver of broilers

**Figure 4:**
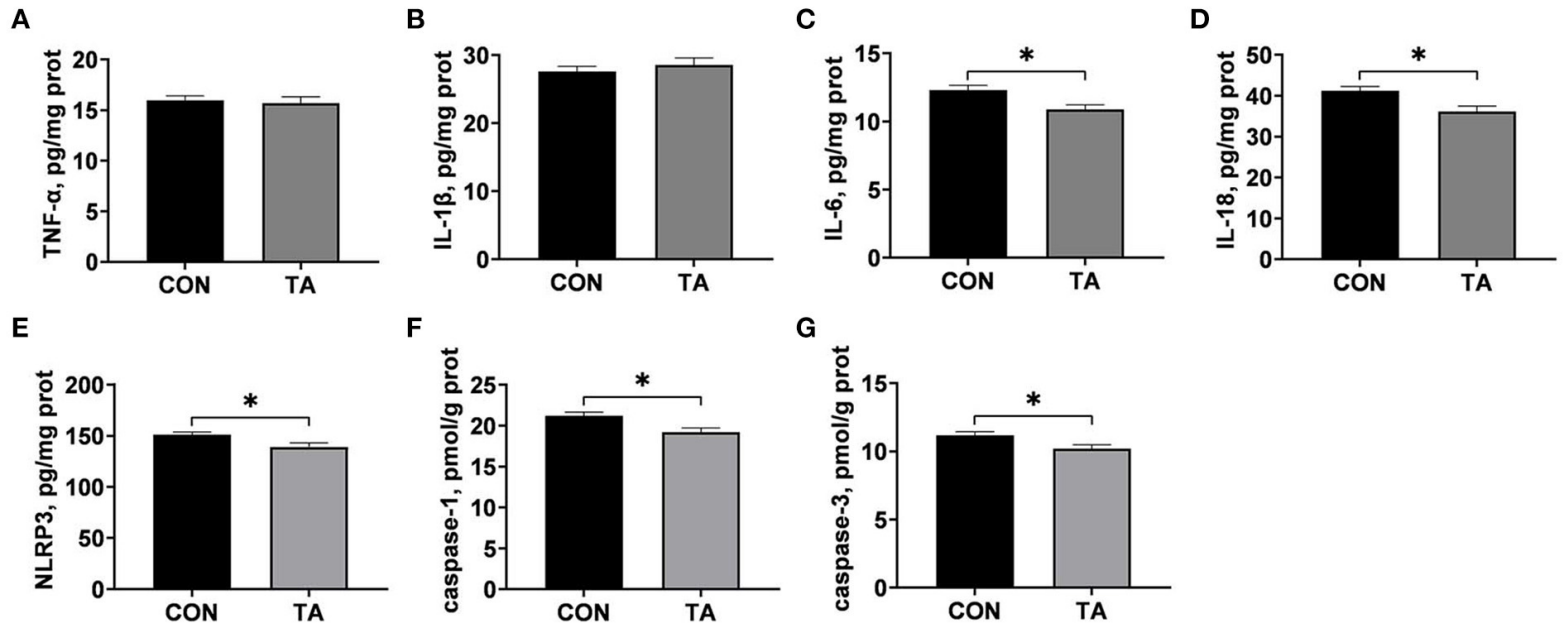
Effects of dietary supplemented with microencapsulated TA from *Galla Chinensis* on liver inflammatory factor and hepatic caspases levels of broiler chickens. **(A)** Tumor necrosis factor-α (TNF-α), **(B)** Interleukin-1β (IL-1β), **(C)** Interleukin-6 (IL-6), **(D)** Interleukin-18 (IL-18), **(E)** NLRs family pyrin domain containing 3 (NLRP3), **(F)** caspase-1, **(G)** caspase-3. CON, broiler chickens fed a basal diet; TA, broiler chickens fed a basal diet supplemented with 300 mg/kg microencapsulated TA from *Galla Chinensis*. Values are mean ± standard error (*n* = 6). *P < 0.05.

Effects of TA on liver inflammatory factors concentrations and hepatic caspases activities of broilers are displayed in Figure 4. Broilers in TA group had significantly lower (*P* < 0.05) levels of IL-6, IL-18, NLRP3, caspase-1, and caspase-3 in livers than those in CON group. There were no significant differences (*P* > 0.05) in liver TNF-α and IL-1β concentrations between the two groups.

### Effects of TA on genes expressions in livers of broilers

**Figure 5:**
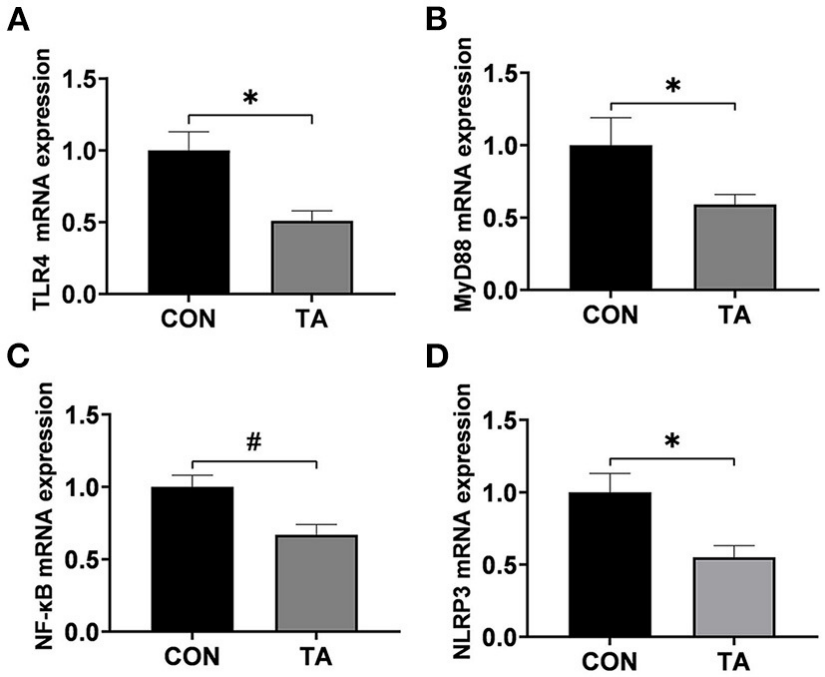
Effects of dietary supplemented with microencapsulated TA from *Galla Chinensis* on inflammatory genes in live of broiler chickens. **(A)** Toll-like Receptor 4 (*TLR4*), **(B)** Myeloid differentiation primary response 88 (*MyD88*), **(C)** Nuclear factor-kappa B (*NF-*κ*B*), **(D)** NLRs family pyrin domain containing 3 (*NLRP3*). CON, broiler chickens fed a basal diet; TA, broiler chickens fed a basal diet supplemented with 300 mg/kg microencapsulated TA from *Galla Chinensis*. Values are mean ± standard error (*n* = 6). *P < 0.05, ^#^0.05 < *P* < 0.1.

The mRNA expression levels of inflammatory genes in liver are presented in Figure 5. Compared with the CON group, the mRNA expressions levels of *TLR4, MyD88*, and *NLRP3* were significantly lower (*P* < 0.05) in the TA group, and the mRNA expression levels of *NF-*κ*B* tended to decease (*P* < 0.10) in the TA group.

**Figure 6:**
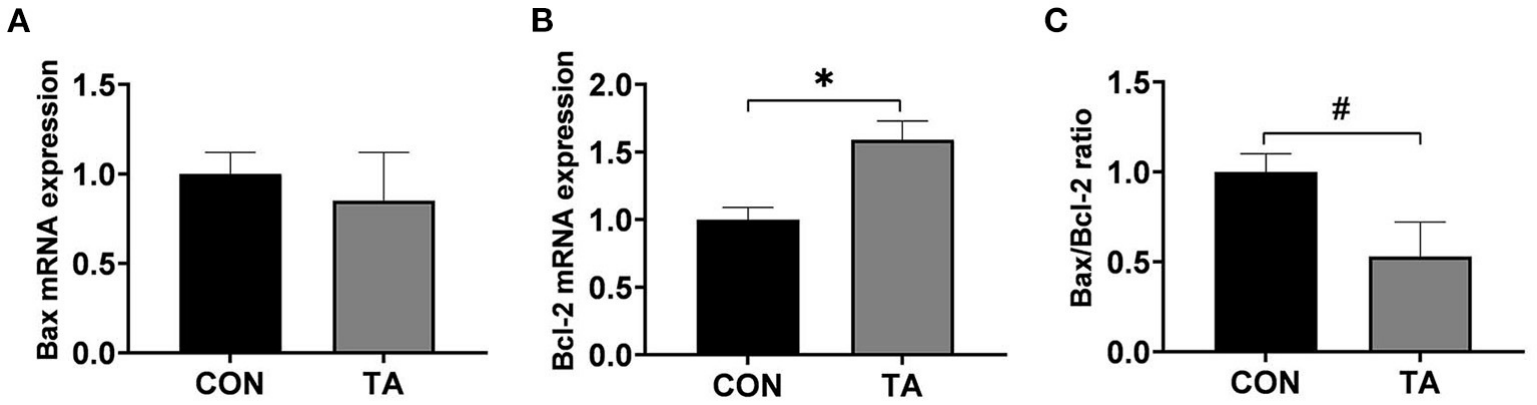
Effects of dietary supplemented with microencapsulated TA from *Galla Chinensis* on apoptosis-related genes in live of broiler chickens. **(A)** BCL2-Associated X (Bax), **(B)** B-cell lymphoma-2 (Bcl-2), **(C)** Bax/Bcl-2 ratio. CON, broiler chickens fed a basal diet; TA, broiler chickens fed a basal diet supplemented with 300 mg/kg microencapsulated TA from *Galla Chinensis*. Values are mean ± standard error (*n* = 6). *P < 0.05, ^#^0.05 < *P* < 0.1.

The mRNA expression levels of apoptosis regulators are shown in Figure 6. The mRNA expression level of *Bcl-2* in liver was significantly lower (*P* < 0.05) in the TA group than in the CON group, and the *Bax/Bcl-2* ratio in the TA group tended to be decreased (*P* < 0.10) than that in the CON group. No significant difference was observed (*P* > 0.05) in the mRNA expression levels of *Bax* in liver between the CON group and the TA group.

## Discussion

In the present study, we found that dietary supplemented with 300 mg/kg microencapsulated TA from *Galla Chinensis* increased final BW and decreased the F/G in broilers throughout the trial. Similarly, Masek, et al. (30) showed that dietary supplemented with 5 g/kg TA increased final BW and ADG, and decreased the F/G of broilers. It was also reported that supplementing 500 mg/kg TA alone increased F/G in coccidiosis-vaccinated broilers (31). Another study in piglets showed that dietary *Galla Chinensis* TA supplementation benefited to the growth performance (32). However, Ebrahim, et al. (33) showed that 1% TA in the diet reduced feed intake of broiler chicken, which might be related to the poor palatability of TA. Previous study indicated that microencapsulation was a potential scheme to improve the palatability in animal feed, and could avoid the undesirable irritation of TA (22). Above all, the results suggested that dietary supplemented with 300 mg/kg microencapsulated TA from *Galla Chinensis* had the potential to improve the growth performance of broilers.

Immune function is an important factor in improving growth performance of broilers (11). The results of this study showed that TA increased serum concentrations of IgG, IgM, C3, and C4. Immunoglobulin is the one of the main anti-infective components of blood (34). As one of the hallmarks of adaptive immunity, immunoglobulins serve as the first line of host defense against infections, and play a vital role in immune regulation and immunological tolerance (35). However, Marzo, et al. (36) demonstrated that adding 25 or 30 g/kg TA to the diet reduced total IgM and IgA levels in blood, which might be due to the high tannin contentions in the diet. Besides, the complement system is an ancient and evolutionary conserved element of the innate immune mechanism, and plays a crucial role in tissue homeostasis and pathogen immunosurveillance (37). The increased immunoglobulin concentrations could also stimulate the increase of complement concentrations and produce a specific immune mechanism (38). In addition, liver is one of the main sites of complement synthesis, serum complement C3 will decrease correspondingly when the liver lesions occur (39). The increased complement C3 and C4 in TA group also suggested that TA benefited to liver protection. To sum up, our study indicated that 300 mg/kg microencapsulated *Galla Chinensis* TA supplementation enhanced the immune function in liver of broilers.

Serum metabolites could indirectly reflect the physiological, health status, and material metabolism status of animals (40). Our current study showed that *Galla Chinensis* TA supplementation increased serum TP, ALB, HDL, LDL, UREA, TCHO, and GLU, and decreased serum ALT activity. The concentrations of TP and ALB in serum reflect the metabolism and nutritional status of protein, and serum TP and ALB are the raw materials of immunoglobulin synthesis (41). Previous study in buffalo showed that dietary TA increased the serum levels of TP and ALB, but did not impact serum concentrations of TCHO and UREA (42). These results indicated that TA could promote protein synthesis, which was beneficial to the growth and immunity of broilers. Previous studies showed that LDL promotes fat deposition, while HDL reduced fat deposition and transfers fat around the liver (43). Yugaran, et al. (44) found that feeding TA increased HDL in serum of rats. The levels of UREA and GLU reflect the metabolic ability of the body (45). Liu, et al. (11) found that the vigorous metabolism of broilers may lead to the increase of serum metabolites of broilers. Therefore, in our study, the increase in serum metabolite concentrations of broilers in TA group may be due to the improvement of growth performance and metabolism of broilers by *Galla Chinensis* TA. Alanine transaminase is a major hepatic transaminase, and ALT will be released into the bloodstream when the liver is damaged (46). Chu, et al. (47) found that TA could reduce the concentrations of ALT in serum of rat carbon tetrachloride-induced liver injury. Based on this, dietary 300 mg/kg microencapsulated TA from *Galla Chinensis* supplementation benefited liver health of broilers.

In order to clarify the underlying mechanism of TA protecting liver, inflammatory factors levels and TLR4/MyD88/NF-κB signaling pathway were assessed in this study. We found that dietary *Galla Chinensis* TA supplementation reduced the levels of IL-6, IL-18, and NLRP3 in liver of broilers. The proinflammatory cytokines IL-6 and IL-18 have critical roles in establishment of inflammation, and involved in the induction of liver diseases (48, 49). Yin, et al. (50) also found that diets supplemented with 500 mg/kg TA decreased serum IL-6 contention in broilers. The inflammasome NLRP3 plays a key role in a variety of disease processes (51). Studies had shown that NLRP3 could activate caspase-1, leading to the maturation of IL-1β and IL-18 and cell necrosis (52, 53). Sun, et al. (54) found that *Galla Chinensis* could significantly reduce the levels of proinflammatory cytokines. These results showed that supplemented with *Galla Chinensis* TA reduced the secretions of inflammatory cytokines in liver of broilers. Besides, the TLR4/MyD88/NF-κB signal mainly mediates the inflammatory response, and is a signal transduction pathway regulating liver diseases (55). In this study, we found that the mRNA expression levels of *TLR4, MyD88, NF-*κ*B*, and *NLRP3* were lower in the TA group than in the CON group. The NF-κB is a key transcription factor mediating inflammatory response and is a prerequisite for the activation of NLRP3 inflammasome (56–58). Studies have shown that TLR4 is a transmembrane protein and belongs to the pattern recognition receptor family, and can also participate in the conduction of inflammatory signals, leading to the release of inflammatory factors such as IL-6, IL-1β, and TNF-α (59, 60). Besides, MyD88, a key adaptor protein of TLR4, and can activate NF-κB and stimulate the secretion of proinflammatory cytokines (61). Previous studies indicated that inhibiting the TLR4/MyD88/NF-κB signaling pathway benefited to attenuate hepatic inflammatory injury (62). Many researches also revealed that TA could inhibit the expression of TLR4 and NF-κB to suppress the inflammatory response in inflammatory cells (63, 64). Therefore, our results suggested that the addition of 300 mg/kg *Galla Chinensis* TA could inhibit hepatic inflammatory response through TLR4/MyD88/NF-κB signaling pathway.

Hepatocyte cell death, as well as liver inflammation, had been well–recognized as central characteristics of liver disease (65). In present study, dietary 300 mg/kg TA supplementation was found to decrease the activities of caspase-1 and caspase-3 and Bax/Bcl-2 ratio, and increase the mRNA expression levels of *Bcl-2* in liver of broiler. Caspases are a family of endoproteases, and activation of caspases ultimately results in programmed execution of cell death including cell apoptosis and pyroptosis (66). Caspase 1 and caspase 3 have been shown to be involved extensively in the apoptotic process, caspase-1 known as IL-1β-converting enzyme can be activated by various inflammasomes such as NLRP3, resulting in pyroptosis of damaged cells (67). Besides, mature caspase-1 effector molecules precaspase-1 can induce IL-1β and IL-18 maturation, leading to cell necrosis (68). Caspase-3, onto which there is a convergence of the extrinsic and intrinsic apoptotic pathways, is the main executioner of apoptosis (69). Activation of caspase-3 was reported to mediate proteasome inhibitor-induced apoptosis (70). Anti-apoptotic Bcl-2 could inhibit apoptosis induced by a variety of factors and play an important role in regulating the apoptotic response (71). The Bax/Bcl-2 ratio has been shown to serve as a check point for the death, and a high Bax/Bcl-2 ratio was confirmed to increase activation of caspases including caspase-1 and caspase-3 (24, 72). Ji, et al. (73) found that *Galla Chinensis* increased Bcl-2 protein expression and decreased epithelial cell damage in patients with pneumonia. Previous studies had shown that TA reduced mitosis and apoptosis in the caecum and descending colon of piglets (74). Inflammation is a potent inducer of cell death through increasing the Bax/Bcl-2 ratio and activating caspases (75). Above all, it demonstrated that the addition of 300 mg/kg microencapsulated *Galla Chinensis* TA could decrease hepatic apoptosis and pyroptosis partially *via* inhibiting inflammation.

## Conclusion

In conclusion, dietary supplemented with 300 mg/kg microencapsulated *Galla Chinensis* TA had beneficial effects on growth performance, immune function, and liver health status in broilers. The protective role of TA in liver health of broilers might be related to the inhibition of hepatic apoptosis and pyroptosis *via* inactivation of TLR4/MyD88/NF-κB signaling pathway. These findings provided new insights into the use of microencapsulated TA from *Galla Chinensis* as a new feed additive in poultry production.

## Data availability statement

The original contributions presented in the study are included in the article/supplementary material, further inquiries can be directed to the corresponding authors.

## Ethics statement

The animal study was reviewed and approved by the Protocol for this Experiment Involving Animals was in Accordance with the Guidelines of the Care and Use Committee of Shandong Agricultural University (Ethics Approval code: SDAUA-2021-019).

## Author contributions

HL and YLi: conceptualization. JN and YLiu: data curation. JN, QW, CJ, and WY: formal analysis. NJ and LH: funding acquisition. CJ and QG: investigation. CJ, HL, and YLi: methodology. JN, QW, YLiu, and YLi: project administration. JN, QW, and HL: software. YLi and WY: supervision. SJ and YLi: validation. JN: writing—original draft. QW, YLi, and WY: writing—review and editing. All authors contributed to the article and approved the submitted version.

## Funding

This research was funded by the Shandong Province Pig Industry Technology System (SDAIT-08-04 and SDAIT-08-05) and Postdoctoral Science Foundation of Shandong Agricultural University, grant number 040/76598.

## Conflict of interest

Author QW is employed by Shandong Wonong Agro-tech Group Co., Ltd. Author QG is employed by Shandong Landoff Biotechnology Co., Ltd. The remaining authors declare that the research was conducted in the absence of any commercial or financial relationships that could be construed as a potential conflict of interest.

## Publisher's note

All claims expressed in this article are solely those of the authors and do not necessarily represent those of their affiliated organizations, or those of the publisher, the editors and the reviewers. Any product that may be evaluated in this article, or claim that may be made by its manufacturer, is not guaranteed or endorsed by the publisher.
